# Impact of Type 2 Diabetes Mellitus on Periodontitis-Related Trabecular Bone Changes Assessed by Fractal Analysis: A Comparative Study

**DOI:** 10.7759/cureus.113426

**Published:** 2026-07-26

**Authors:** Rashmi Venkatesh, Radhika S Patel, Chandramani B More, Deepa J Patil, Palak H Shah

**Affiliations:** 1 Oral Medicine and Radiology, K. M. Shah Dental College and Hospital, Sumandeep Vidyapeeth (Deemed to be University), Vadodara, IND; 2 Oral Medicine and Radiology, Manubhai Patel Dental College and Hospital, Vadodara, IND

**Keywords:** alveolar bone, dental radiograph, fractal dimension, periodontitis, type 2 diabetes mellitus

## Abstract

Background

Conventional radiographic imaging often struggles to detect early bone issues, as in early periodontitis, since radiographically detectable changes usually don't appear until a significant amount of bone mineral has been lost. Periodontitis and type 2 diabetes mMellitus (T2DM) have a well-established bidirectional association. This study aimed to assess and compare the trabecular bone structure among healthy individuals, patients with periodontitis, and those with T2DM and concomitant periodontitis using fractal analysis (FA) of digital periapical radiographs.

Materials and methods

The study comprised a total of 120 participants, divided into three equal groups: 40 periodontally healthy individuals, 40 individuals diagnosed with Stage II/III periodontitis, and 40 individuals with T2DM and concurrent Stage II/III periodontitis. The fractal dimension (FD) of the trabecular bone was assessed in both the interdental space and apical region of the mandibular first molar using the box-counting method via ImageJ software.

Results

Statistical analysis revealed a significant difference in FD values of the interdental bone region of interest (ROI) across all three groups (P < 0.05). In contrast, the FD values of the apical ROI did not differ significantly among the groups (P > 0.05).

Conclusion

FD values showed a reduction in the proximal/interdental alveolar bone, with no significant changes in the apical region. Notable differences in FD values among healthy individuals, patients with periodontitis, and those with T2DM and periodontitis suggest an effect of glycemic status on variations in trabecular bone quality.

## Introduction

Periodontitis is a chronic inflammatory condition affecting the supporting structures of the teeth, primarily caused by specific pathogenic microorganisms or microbial complexes. It is typically marked by extensive degradation of the periodontal ligament (PDL) and resorption of the alveolar bone. The primary etiological factor in periodontitis is bacterial plaque, which contains a diverse array of pathogenic microorganisms. Bacterial byproducts, including endotoxins and lipopolysaccharides, play a pivotal role in initiating and sustaining the inflammatory response. Periodontitis manifests with gingival inflammation, periodontal attachment loss, and interdental bone loss [1, 2]. Diagnostic evaluation involves clinical examination, with periodontal probing used to assess attachment loss and the extent of periodontal tissue destruction. Radiographic imaging plays a vital role throughout various stages of periodontal assessment [3].

Type 2 diabetes mellitus (T2DM) is characterized by insulin resistance and progressive impairment of pancreatic beta-cell function, resulting in chronic hyperglycemia. Periodontitis is recognized as the sixth major complication of diabetes mellitus (DM), reflecting the bidirectional relationship between systemic metabolic dysfunction and oral health. Blood glucose concentration, particularly elevated levels associated with diabetes, plays a significant role in periodontal collagen turnover. High blood sugar impairs collagen production and increases its breakdown, contributing to periodontal disease. In addition, compromised neutrophil function in individuals with DM leads to heightened periodontal tissue destruction. Also, periodontal disease can interfere with blood sugar management, potentially leading to uncontrolled diabetes by worsening blood sugar control [4-5]. In individuals with diabetes, the trabecular bone score (TBS), which evaluates bone microarchitecture, is often diminished, indicating reduced bone quality even when bone mineral density (BMD) appears normal or elevated. This phenomenon, known as the "diabetic paradox," underscores the increased fracture risk in T2DM patients despite seemingly adequate BMD. Intraoral periapical radiography is employed to monitor the condition of periodontal tissues with precision. Radiographic detection of bone changes typically requires resorption of at least 30% of the bone's mineral content, limiting the sensitivity of conventional imaging in early diagnosis. However, with the help of newer techniques, trabecular microarchitecture can be studied [6-9].

Fractal analysis (FA) of trabecular bone texture quantifies the complexity of a radiographic pattern. It helps distinguish between healthy and osteoporotic bone and can be a complementary tool to BMD measurements. Fractal dimension (FD) analysis is a technique used to detect intricate structural patterns within trabecular bone, providing a quantitative measure of its complexity. When applied to periapical radiographs, FD effectively reflects the structural intricacy of the alveolar bone surrounding the teeth. Although various techniques exist for estimating FD, the White and Rudolph box-counting method remains the most widely adopted, particularly due to its suitability for analyzing binary images. Numerous studies have investigated trabecular alterations in the alveolar bone of patients with periodontitis, consistently demonstrating a decline in FD correlating with the progression of the disease [10-13]. However, few studies have independently evaluated FD values in periodontitis patients with and without T2DM. Therefore, the present study aims to assess radiographic trabecular complexity in periapical radiographs of T2DM patients with periodontitis, comparing them to systemically healthy individuals with periodontitis using FA.

## Materials and methods

Ethical statement 

The present study received approval from the Sumandeep Vidyapeeth Institutional Ethical Committee (SVIEC/RP/May/24/56 dated 21/05/2024). The study was conducted in the Department of Oral Medicine and Radiology at K. M. Shah Dental College and Hospital, Vadodara, India, between June 1, 2024 and June 30, 2025. Written informed consent was obtained from each participant in either English or their respective vernacular language. The study was conducted in accordance with the principles outlined in the Declaration of Helsinki [14].

A priori sample size calculation was performed to ensure adequate statistical power. A total of 120 participants were enrolled and categorized into three groups. Group I comprised 40 patients diagnosed with Stage II and Stage III periodontitis. These individuals had no systemic disorders and were not on any medications or supplements. Only vital mandibular molars were considered, excluding teeth with endodontic lesions, prior endodontic treatments, or restorative crowns. Group II included 40 patients with T2DM and concomitant periodontitis (stage II and III). Potential confounding variables such as smoking status, osteoporosis, medication history, and duration of diabetes were considered during participant screening. Participants had no other systemic conditions, exhibited moderate glycemic control (HbA1c levels between 7% and 8%), and presented with endodontically healthy mandibular molar teeth. Group III consisted of 40 healthy controls matched for age and sex, with endodontically healthy mandibular molar teeth and no periodontitis or any systemic disease.

All periodontal assessments and radiographic evaluations were conducted by a single calibrated examiner to ensure consistency. Digital periapical radiographs of the mandibular first molar were acquired as part of routine clinical care using the paralleling technique with size 2 imaging plates (Dürr Dental) and standard posterior film holders to ensure optimal image quality and reproducibility. All radiographs were captured using an intraoral X-ray unit (Carestream CS2100) set to standardized exposure parameters of 60 kVp, 7 mA, and an exposure time of 0.2 seconds. Patients exhibiting clinical attachment loss (CAL) of 3-4 mm along with radiographic bone loss (RBL) confined to the coronal third of the root, approximately 15% to 33%, were classified as having Stage II periodontitis. Those with CAL greater than 5 mm and RBL extending to the middle third of the root or beyond were categorized as having Stage III periodontitis [15]. Mandibular first molars were selected as they are commonly affected in periodontitis and typically exhibit early and progressive periodontal changes, making them ideal for evaluating alveolar bone microarchitecture. Healthy individuals with no evidence of CAL were included in the control group.

**Figure 1:**
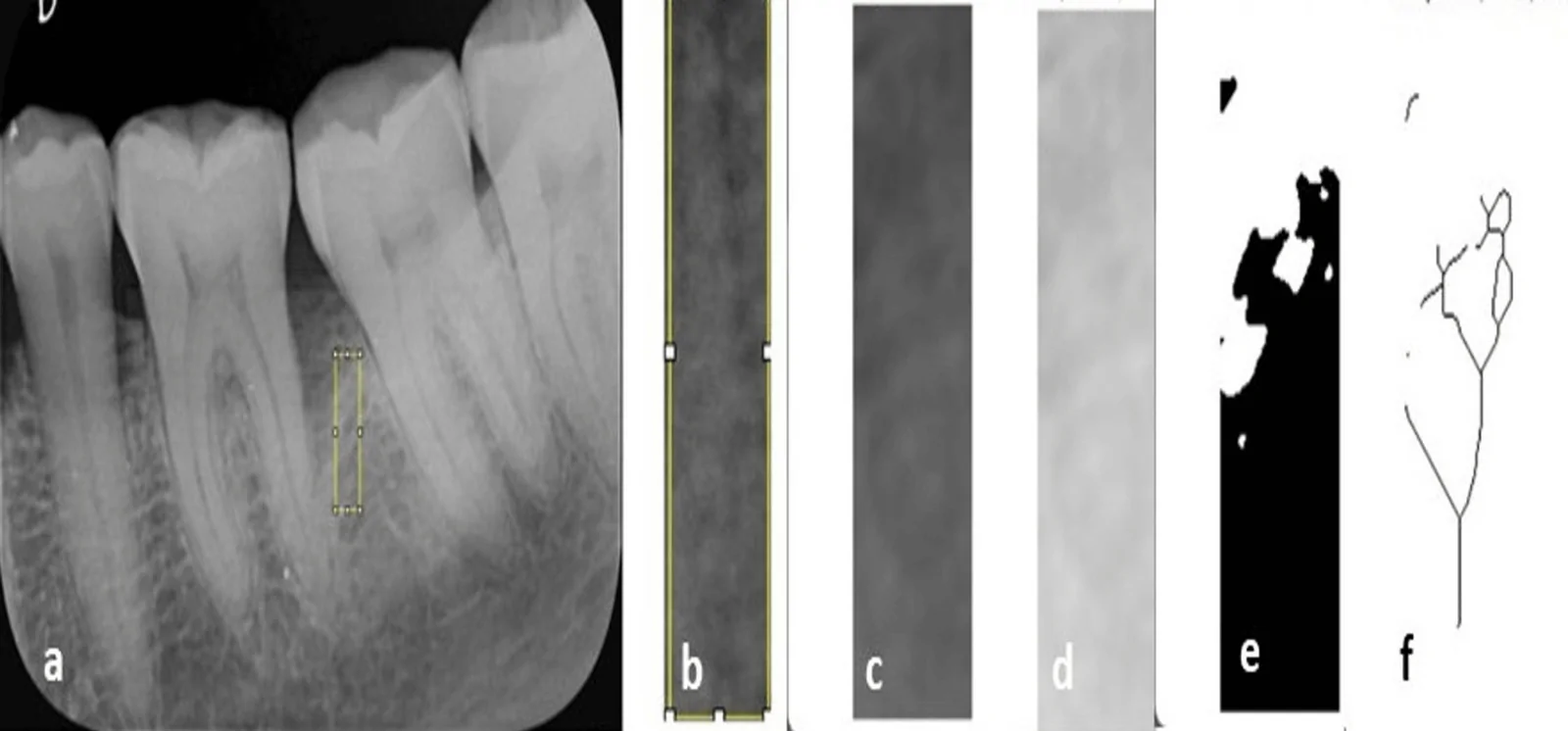
Fractal dimension (FD) analysis using the box-counting method in ImageJ. (a) Selection of regions of interest (ROIs) used to calculate the fractal dimension using ImageJ software. (b-f) Processing of the ROI for FD calculation: (b) duplicated ROI, (c) Gaussian blur filtering, (d) image after subtraction and addition of 128 gray values, (e) binary conversion and inversion, and (f) skeletonization.

**Figure 2:**
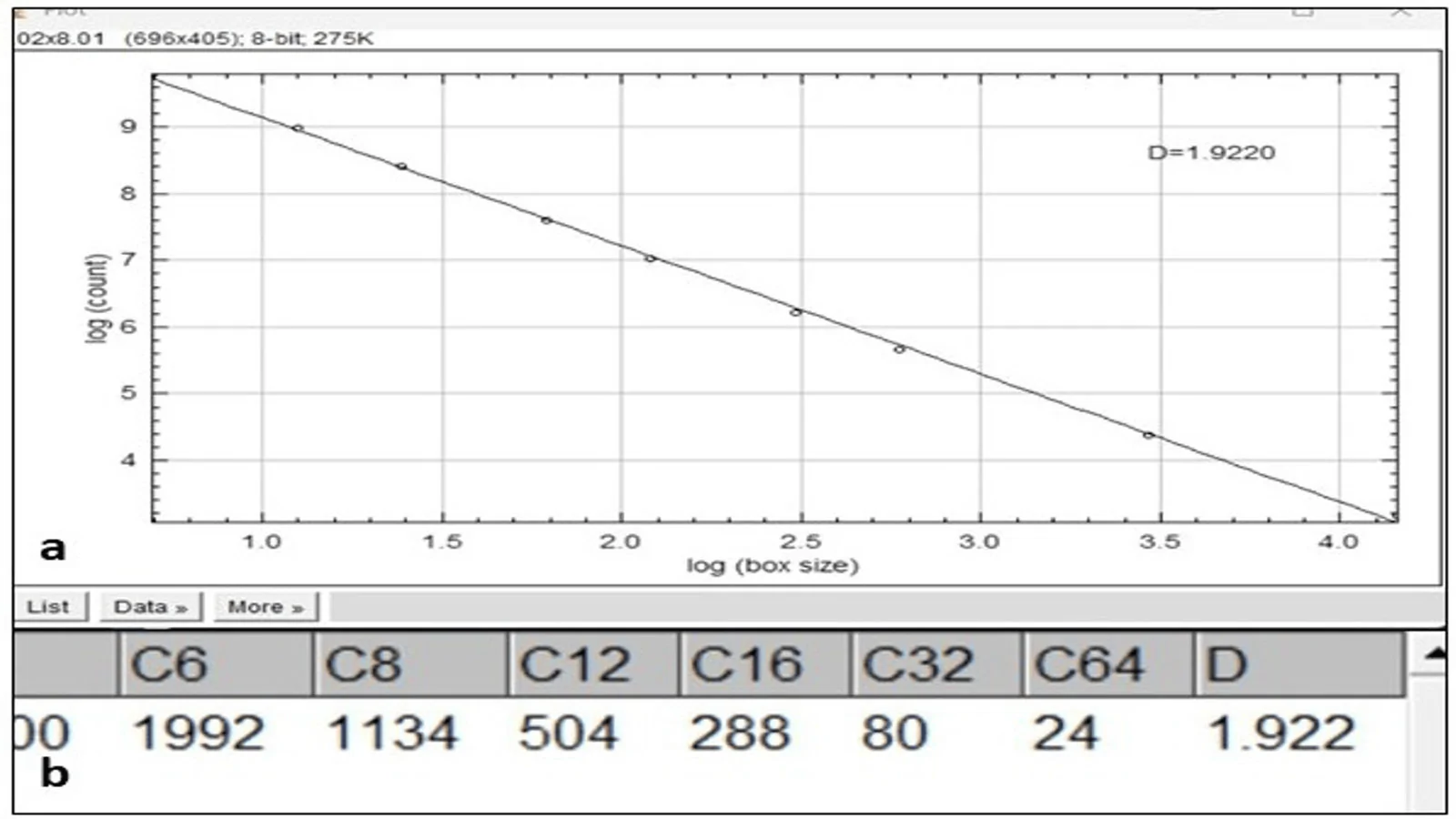
Fractal dimension (FD) analysis results using the box-counting method in ImageJ. (a) Graph showing a linear relationship. (b) Results table displaying quantitative measurements of FD.

For FD analysis, the DICOM files of the digital radiographs were exported to ImageJ software (National Institutes of Health, USA). Examiner calibration was undertaken, and intra- and inter-observer reliability were assessed to minimize variability in measurements. In each radiograph, three regions of interest (ROIs) were selected: apical, mesial, and distal areas of the mandibular first molar. Mesial and distal ROIs were defined as rectangular areas extending from the alveolar crest to a line connecting the apices of the roots. The apical ROI was selected as a horizontal rectangle encompassing the apical region of both roots. Each ROI was isolated from the original image, and the extracted regions were then duplicated. A Gaussian blur filter (sigma = 10) was applied to the duplicate image to minimize noise. The blurred image was subtracted from the original to enhance structural details, followed by conversion to binary format using the “Make Binary” tool. The mean intensity threshold was set at 128 for 8-bit images. Finally, the “Fractal Box Count” tool was employed to calculate the FD of each ROI (Figures 1-2).

Statistical analysis 

Quantitative data were presented as mean value ± SD. The distribution of data was assessed using the Kolmogorov-Smirnov test. Statistical analysis was performed using SPSS, v. 20, with the chi-square test, one-way ANOVA, and Tukey’s post hoc test for multiple comparisons (p < 0.05).

## Results

Periapical radiographs were obtained from a total of 120 individuals, categorized into three groups: 40 patients with periodontitis (30 with Stage II and 10 with Stage III), 40 patients diagnosed with T2DM and concomitant periodontitis (24 with Stage II and 16 with Stage III), and 40 age- and sex-matched healthy controls. Age and sex distribution was not statistically different among the groups. Among the patients with T2DM and periodontitis, glycemic control was classified as moderate. An overview of the participants' demographic characteristics is provided in Table 1.

**Table 1:** Demographic characteristics of the study participants. T2DM: Type 2 Diabetes mellitus.

Groups	Number of Participants n (%)			Age (Mean ± SD)	Periodontitis Stage		
	Males	Females	Total		Stage II	Stage III	Chi-square test
Healthy controls	21 (52.5)	19 (47.5)	40 (100)	48.93 ± 8.05	-	-	-
Periodontitis	21 (52.5)	19 (47.5)	40 (100)	46.35 ± 8.61	30 (75)	10 (25)	Chi-square value = 2.051; P = 0.152
T2DM with periodontitis	21 (52.5)	19 (47.5)	40 (100)	46.55 ± 8.37	24 (60)	16 (40)	

Patients in the periodontitis group and the T2DM with concomitant periodontitis group had Stage II (CAL: 3-4 mm, RBL: in the coronal third) and Stage III periodontitis (CAL: ≥5 mm, RBL: in the middle third of the root and beyond). The distribution of patients with Stage II and Stage III periodontitis between both groups was not statistically significant (p-value = 0.028 for mesial interdental bone and p-value = 0.023 for distal interdental bone) (Table 2).

**Table 2:** Comparison of RBL measurements among different study groups. RBL: Radiographic bone level; DM: Diabetes mellitus.

Variables	n	Group	One Way ANOVA Test			Tukey's Post Hoc Test for multiple comparisons		
			Mean ± SD	Source	F-value, p-value	Groups		p-value
RBL Mesial (mm)	40	Healthy controls	1.00 ± 0.00	Between Groups	140.099, <0.001	Control	Periodontitis without DM	<0.001
	40	Periodontitis	2.13 ± 0.33	Within Groups			Periodontitis with DM	<0.001
	40	DM with periodontitis	2.35 ± 0.58	Total		Periodontitis without DM	Periodontitis with DM	0.028
RBL Distal (mm)	40	Healthy controls	1.00 ± 0.00	Between Groups	109.7, <0.001	Control	Periodontitis with DM	<0.001
	40	Periodontitis	2.05 ± 0.39	Within Groups			Periodontitis with DM	<0.001
	40	DM with periodontitis	2.30 ± 0.61	Total		Periodontitis without DM	Periodontitis with DM	0.023

FD values of the interdental bone ROI (mesial and distal) were significantly different among all three groups (P < 0.05); however, the FD values of the apical ROI were not significantly different among the groups (P > 0.05) (Table 3).

**Table 3:** Comparison of mean fractal dimension (FD) values in different regions of interest among the study groups. FD: Fractal dimension; DM: Diabetes mellitus.

Variables	n	Group	One Way ANOVA Test			Tukey's Post Hoc Test for multiple comparisons			
			Mean ± SD	Source	F-value, p-value	Groups		Mean Difference	p-value
FD in Mesial Interdental bone	40	Healthy controls	1.89 ± 0.05	Between Groups	21.483, <0.001	Control	Periodontitis without DM	0.04	0.002
	40	Periodontitis	1.84 ± 0.06	Within Groups			Periodontitis with DM	0.08	<0.001
	40	DM with periodontitis	1.80 ± 0.06			Periodontitis without DM	Periodontitis with DM	0.04	0.007
FD in Distal Interdental bone	40	Healthy controls	1.86 ± 0.06	Between Groups	7.742, 0.001	Control	Periodontitis without DM	0.02	0.159
	40	Periodontitis	1.84 ± 0.07	Within Groups			Periodontitis with DM	0.06	<0.001
	40	DM with periodontitis	1.81 + 0.07			Periodontitis without DM	Periodontitis with DM	0.03	0.097
FD in Apical bone	40	Healthy controls	1.84 + 0.06	Between Groups	2.834, 0.063	Control	Periodontitis without DM	0.02	0.328
	40	Periodontitis	1.82 + 0.06	Within Groups			Periodontitis with DM	0.03	0.051
	40	DM with periodontitis	1.80 + 0.07			Periodontitis without DM	Periodontitis with DM	0.01	0.622

## Discussion

The non-enzymatic glycation of collagen fibers, an uncontrolled reaction between extracellular sugars and amino acid residues, leads to the accumulation of advanced glycation end-products (AGEs). This process compromises the quality of the bone tissue matrix, contributing to reduced trabecular bone score (TBS) and impaired bone microarchitecture. Individuals with T2DM are significantly more susceptible to periodontitis than their nondiabetic counterparts. AGEs play a pivotal role in the progression of periodontitis by amplifying the inflammatory response and contributing to periodontal tissue destruction [15]. Despite this known association, data on the jawbone’s microarchitectural characteristics in T2DM patients with concurrent periodontitis remain extremely limited. The present study evaluated trabecular bone changes in periapical radiographs of T2DM patients with periodontitis, comparing them to systemically healthy individuals with periodontitis using fractal analysis.

Fractal analysis was performed on the alveolar bone surrounding healthy mandibular first molars, as well as those affected by Stage II and III periodontitis, with or without T2DM. For each tooth, three ROIs were selected: two proximal sites (mesial and distal) and one apical site. The trabecular bone distribution within these ROIs was evaluated using the box-counting method of fractal analysis in ImageJ software.

Fractal analysis of the proximal regions of the mandibular first molar revealed significant differences in cancellous bone structure, thereby rejecting the null hypothesis. The FD values effectively distinguished between healthy individuals and those with Stage II and III periodontitis, with or without T2DM. Notably, fractal analysis detected progressive bone alterations across the groups, with FD scores decreasing in the following order: healthy controls, periodontitis patients, and individuals with both periodontitis and T2DM. These differences were statistically significant, underscoring the sensitivity of fractal analysis in identifying microstructural changes in alveolar bone. Consistent with our findings, previous studies employing FD analysis have demonstrated significant differences in mean FD values between healthy individuals and those at various stages of periodontitis [10-12].

The current study revealed a significant reduction in FD scores among patients with T2DM and periodontitis, highlighting a pronounced deterioration in trabecular bone architecture. In contrast to our study results, a prior investigation reported lower fractal analysis values in individuals with periodontitis compared to healthy controls [16]. However, their findings did not indicate any diabetes-related alterations in bone trabeculation associated with periodontitis. The present study lacked a T2DM-only cohort, whether well controlled or poorly controlled, and therefore cannot attribute reduced FD independently to diabetes. A study conducted in Turkey utilized cone-beam computed tomography (CBCT) to assess FD values in the basal bone of the posterior mandibular region, located superior to the mandibular canal, in both T2DM patients and healthy controls. However, the participants in both groups did not demonstrate periodontitis. The authors reported no statistically significant differences in FD values between the two groups [8]. However, numerous other studies have documented an increased incidence of fragility fractures in individuals with T2DM, affecting sites such as the vertebrae and hip. Additionally, correlations between BMD and osteoporosis have been observed in the radius, lumbar spine, and hip bones of T2DM patients. Research has demonstrated that individuals with T2DM face a heightened risk of fractures, even when their BMD is normal or elevated. This paradoxical increase in fracture susceptibility is thought to stem from disruptions in bone microarchitecture and/or compromised bone quality, particularly in the properties of the bone matrix [7].

Women typically begin to exhibit signs of osteoporosis during the perimenopausal period. Age-related osteoporotic changes are well documented in the literature, with particular emphasis on the progressive thinning of the cortical bone layer, which can be assessed using various techniques, such as dual-energy X-ray absorptiometry (DXA) parameters and biochemical markers [17-22]. The primary objective of the present study was to assess the quality of alveolar bone trabeculation in individuals with periodontitis. Notably, reduced FD values were observed in the proximal bone of patients with T2DM who also presented with periodontitis, indicating compromised trabecular bone structure.

Limitations of the study

The present study was limited by its small sample size and single-center design. FD values could also be assessed and compared between patients with periodontitis only and patients with diabetes only, incorporating extended follow-up periods instead of a cross-sectional design for a more robust analysis.

## Conclusions

In conclusion, our findings revealed a reduction in FD values within the proximal/interdental alveolar bone, with no significant changes in the apical region, and demonstrated notable variation in FD values among healthy individuals, those with periodontitis, and patients with both T2DM and periodontitis, indicating differential trabecular bone quality across these groups. However, the cross-sectional design cannot establish causality, progression, or a diabetes-specific effect on the trabecular pattern in periodontitis patients. Future research involving larger sample sizes and incorporating biochemical markers such as AGEs and glycated hemoglobin (HbA1c) levels may provide deeper insights into the relationship between systemic conditions and alveolar bone microarchitecture.
